# TFEB-associated renal cell carcinoma: A case report and literature review

**DOI:** 10.1097/MD.0000000000031870

**Published:** 2022-12-16

**Authors:** Yong Zhu, Chengxing Xia, Yitian Ou, Chao Zhang, Lin Li, Delin Yang

**Affiliations:** a Department of Urology, The Second Affiliated Hospital of Kunming Medical University, Kunming, Yunnan, China; b Department of Oncology, Qujing First People’s Hospital, Qujing, Yunnan, China.

**Keywords:** associated renal cell carcinoma, renal cell carcinoma, TFEB

## Abstract

TFEB-associated renal cell carcinoma is very rare and belongs to the microphthalmia — associated transcription family translocation renal cell carcinoma.

**Patient concerns:**

Hospitalized for fever, a 29-year-old male patient had a left kidney lesion without any additional discomfort.

**Diagnoses:**

Histopathological and immunohistochemical results were corresponding with TFEB renall cell carcinoma features.

**Interventions:**

Surgical resection of the tumor was performed.

**Outcomes:**

After 8 months of follow-up, no tumor recurrence was observed.

**Lessons:**

TFEB-associated renal cell carcinoma is rare. The diagnosis is explicit. However, the optimal treatment needs to be further explored.

## 1. Introduction

Various subtypes of renal cell carcinoma (RCC) have distinct histological characteristics, gene mutations, and clinical characteristics. In 2016, a new subtype, known as microphthalmia-associated transcription (MIT) family translocation renal cell carcinoma, was added to the WHO classification of renal cell carcinoma.^[1]^ Renal cell carcinoma, which is composed of 4 transcription factors, microphthalmia — associated transcription factor (MITF), TFEC, TFEB, and TFE3, has been identified as an independent entity known as MITF translocation carcinoma. The latter 2 are associated with the development of renal cell carcinoma. include TFEB-related renal cell carcinoma and renal cell carcinoma linked to Xp11.2 translocation.^[2]^ This study evaluated the pertinent literature and described a case of renal cell cancer linked to TFEB.

## 2. Case report

**Figure 1.**
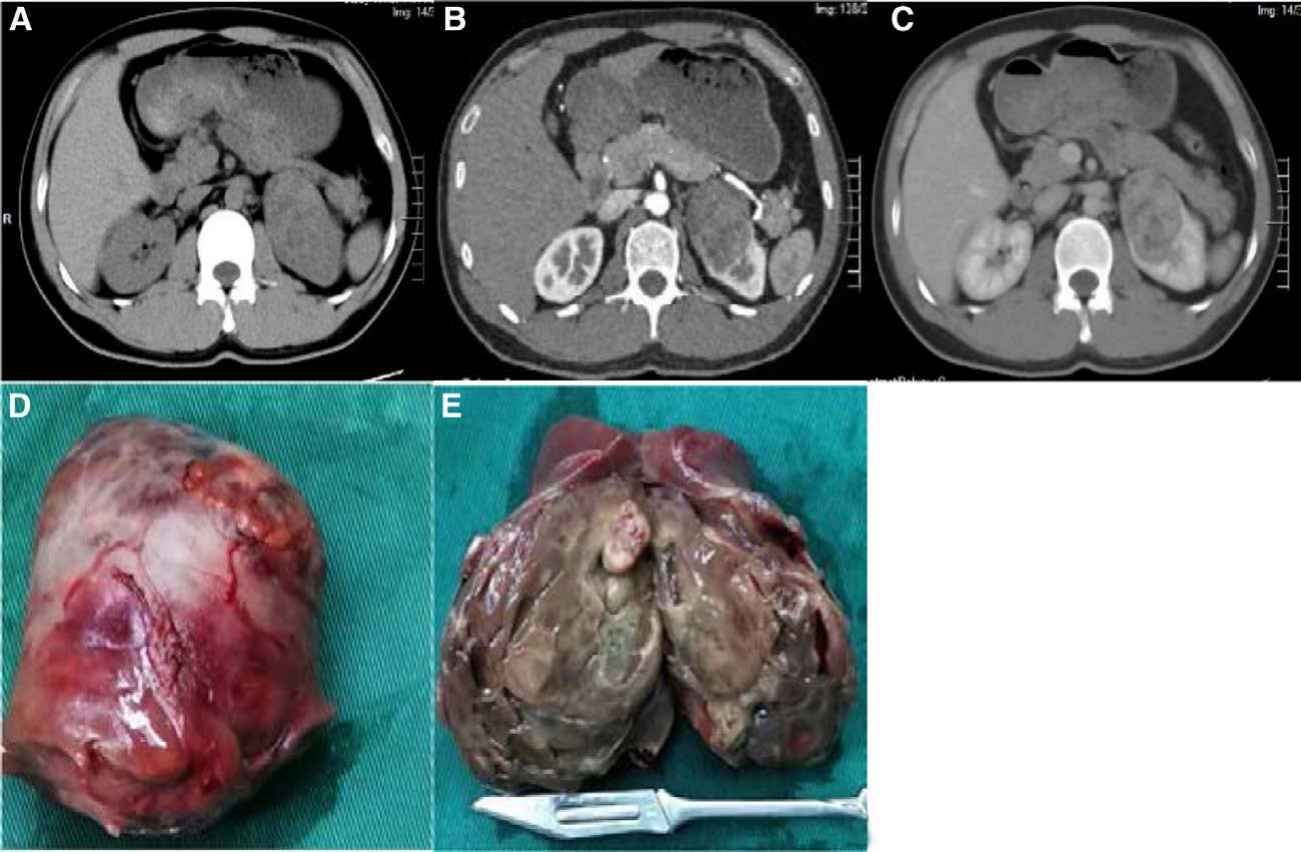
(A) Computed tomography (CT) showing a round mass in the upper left kidney on plain scan. (B and C) Contrast-enhanced CT showing uneven mild enhancement of the tumor, no abnormal density, and enhancement of the right kidney. (D and E) The internal tumor was grayish-yellow, grayish-red, soft, and cystic locally. CT = computed tomography.

**Figure 2.**
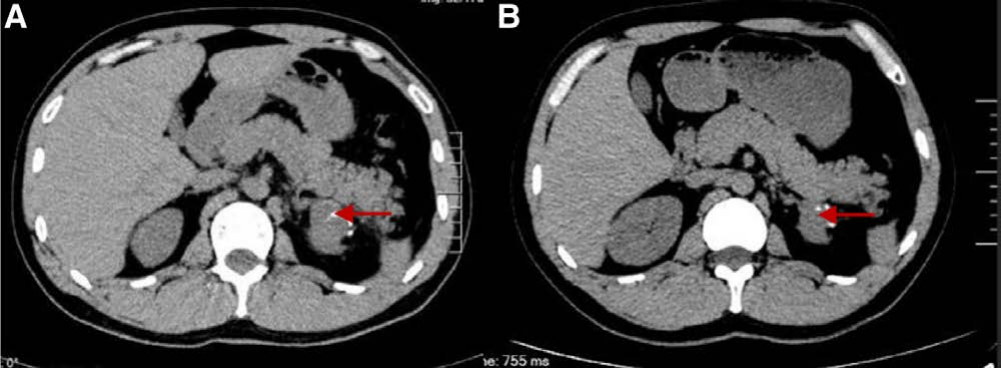
(A) A low-density shadow on the red arrow of the left kidney was seen at 2 months after the operation. (B) The low density of the left kidney was smaller 5 months after the operation.

Hospitalized for fever, a 29-years-old male patient had a left kidney lesion without any discomfort. The physical examination revealed no abnormality. The size, shape, and Color Doppler ultrasonic color imaging results of kidneys were of normal size, and the capsule was smooth. A cystic solid nodule measuring approximately 4.2 * 4.5 cm was found in the upper of the left kidney. Some faint signs of blood flow were observed; however, the boundary was not clear. A computed tomography scan revealed an isodense, spherical mass measuring 5.9 * 4.9 * 6.0 cm in the upper pole of the left kidney (Fig. 1A), Contrast computed tomography scan showed uneven mild enhancement of tumor, no abnormal density, and enhancement of the right kidney (Fig.1B, C). A laparoscopic partial nephrectomy was performed on the left kidney. The postoperative specimens were grayish-white, grayish-brown, and soft. The internal tumor was grayish-yellow, grayish-red, soft, and cystic locally (Fig. 1D, E). The patients recovered well after operation. Adjuvant was not administered. Two months after the surgery, a low-density shadow was observed in the left kidney (Fig. 2A). Five months after the surgery, the low-density shadow in the left kidney became smaller (Fig. 2B). The surgical video was reviewed, and the low-density shadow was considered the necrotic focus of the vascular ligation.

**Figure 3.**
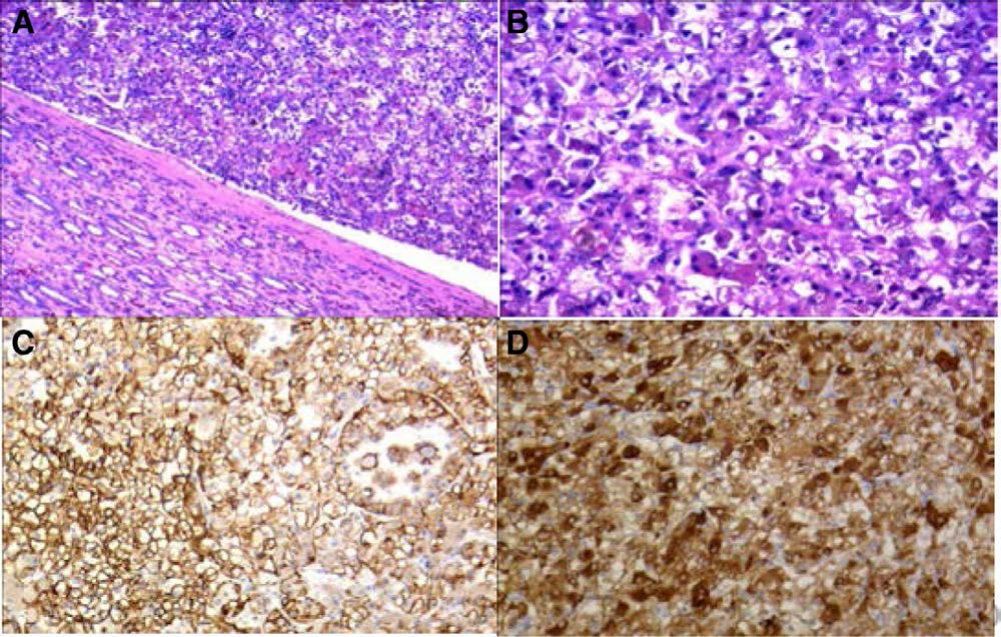
(A and B) Histopathological results were corresponding with TFEB RCC (H&E, A:10 × magnification, B: 20 × magnification), (C)Immunohistochemical staining for CKL was positive (magnification × 20), (D) Immunohistochemical staining for Melan-A was positive (magnification × 20). H&E = hematoxylin and eosin, RCC = renall cell carcinoma.

**Figure 4.**
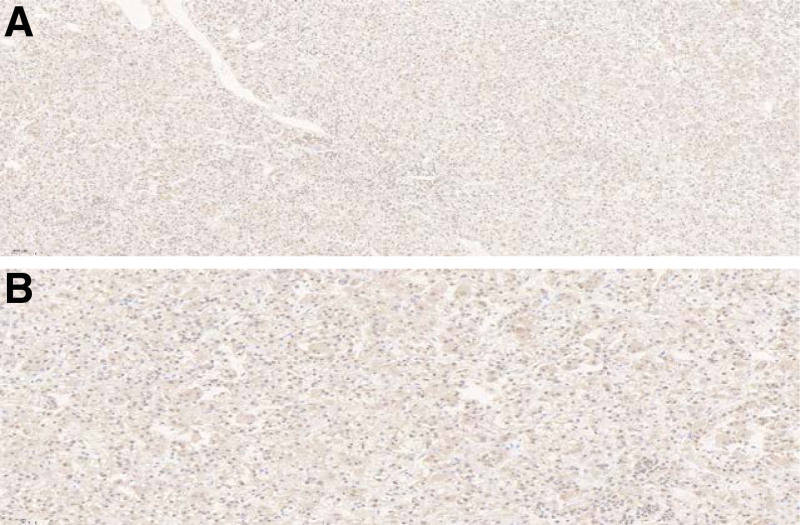
(A) Immunohistochemical staining for TFEB was positive (magnification × 10), (B) Immunohistochemical staining for TFEB was positive (magnification × 20).

Histopathological (Fig. 3A and B) and immunohistochemical results were corresponding with TFEB RCC. In the present study, the tumor cells stained positive forCKL (Fig. 3C), CPAX-8, Pax-2, CD117, P504S, VIM, SDHB, Melan-A (Fig. 3D), E-Cadherin Her-2 and TFEB (Fig. 4A and B), and negative for CD10, MOC-31, CK7, CKH, TFE3, P63, EMA, CAIX, CK20, Myosin, Myogenin, Myo-D1, CK20, CAIX and ALK. The proliferation rate Ki-67 was approximately 10%.

## 3. Discussion

MITF translocation renal cell carcinoma was classified as a separate subtype by the WHO in 2016.^[1]^ MITF translocation renal cell carcinoma has been identified as an independent entity in renal cell carcinoma and consists of 4 transcription factors: MITF, TFEC, TFEB and TFE3. TFEB and TFE3 are associated with sporadic renal cell carcinoma.^[2]^ MITF translocation renal cell carcinoma accounts for about 1/ 3 of ediatric renal tumors. Approximately 15% of patients with renal tumors under 40-years-old have MIT family translocation renal cell carcinoma, and the proportion of adult patients with renal tumors is up to 4%.^[3,4]^ Including Xp11.2 translocation associated renal cell carcinoma and TFEB-associated renal cell carcinoma,^[2,5]^ These partner genes fuse with TFE3 or TFEB genes and activate transcription to further activate downstream genes, resulting in tumorigenesis.^[6,7]^ The TFEB-associated renal cell carcinoma fused alpha (MALAT1) gene and TFEB transcription factor gene result in up regulation of TFEB expression, thereby driving the abnormal expression of melanocyte markers, which is a sign of this unique tumor.^[8]^ For example, Melan-A was expressed in this case. In addition, Zhan et al^[9]^ showed that in cell lines stably transfected with *α*-TFEB, high expression of TFEB enhanced cell invasion, promoted cell proliferation, reduced cell apoptosis, and promoted tumorigenicity in nude mice. TFEB or TFE3 fusion with other partner genes may be the initiating factors for MITF translocation renal cell carcinoma.

TFEB-associated renal cell carcinoma is very rare^[10]^, By 2017, only about 50 cases of TFEB-associated renal cell carcinoma were reported.^[11]^ The main partner gene fused with TFEB is MALAT1 (also known as *α* gene).^[12]^ Other fusion partner genes have recently been described as ACTB, NEAT1^[13]^ and melanin TFEB-associated renal cell carcinoma.^[14]^ Fusion of TFEB translocation with other genes is a common feature of these tumors, and an increasing number of partner genes have been identified. Morphologically, the typical microscopic features of TFEB-associated renal cell carcinoma are biphasic distribution of large epithelioid cells and small cells, eosinophilic cytoplasm of large epithelioid cells, and absence of basement membrane spheres.^[6,15,16]^ In terms of immunohistochemical markers, TFEB-associated renal cell carcinoma was not labeled as an epithelial immunohistochemical marker, but was labeled as melanocyte markers HMB45 and melan-A.^[8]^ It was also reported that HMB45 and melan-A were negative, Sean R. Williamson et al speculated that a subgroup of TFEB-associated renal cell carcinoma lacked melanocyte markers.^[17,18]^ In our reported cases, Melan-A was expressed without HMB45. Because the biphasic structure observed under a microscope, some diagnostic clues can be provided.^[6,15,16]^ Immunohistochemical (IHC) of TFEB protein has diagnostic value in TFEB-associated renal cell carcinoma. However, due to the limitations of staining conditions and techniques, IHC results in false positive or false negative. Fluorescence in situ hybridization (FISH) is the gold standard for current diagnosis.^[19–22]^ One of the differential diagnoses for TFEB-associated renal cell carcinoma is TFEB-amplified renal cell carcinoma. These are all TFEB variants of renal cell carcinoma. FISH detection of TFEB protein is an effective identification method. In addition, IHC for MelanA is also an effective identification method, which is positive in 90 % and 60 % of TFEB-associated renal cell carcinomas and TFEB-amplified renal cell carcinomas, respectively.^[10]^ Another differential point is epithelioid angiomyolipoma, both of which show significant IHC overlap, consistently label melanocyte markers and cathepsin K, usually label CD117 and vimentin. PAX8 is a specific marker for distinguishing TFEB-associated renal cell carcinoma from epithelioid angiomyolipoma. But unfortunately has low sensitivity, as not all TFEB-associated renal cell carcinomas express PAX8.In which case FISH may be an effective method for this distinction.^[23]^ In summary, for TFEB-associated renal cell carcinoma, in the diagnosis and differential diagnosis of translocation renal cell carcinoma and other types of renal cell carcinoma, even between MIT family translocation renal cell carcinoma, morphology, and IHC are good identification methods, and FISH is sometimes necessary.

Surgery is the preferred treatment; however, there are no clear guideline for its treatment.^[24]^ In TFEB-associated renal cell carcinoma, overexpression of mTORC1 induced by TFEB is a key step in kidney, bladder formation and tumorigenesis.^[25]^ Zhang et al^[26]^ found that TFEB binds to PD-L1 promoter in RCC and inhibits mTOR, leading to nuclear translocation of TFEB and enhanced expression of PD-L1. In an RCC xenograft mouse model, inhibition of mTOR and blocking of PD-L1 enhanced CD8 ^+^ cell lysis and tumor suppression. This suggest that immunotherapy and targeted therapy have therapeutic effects in TFEB-associated renal cell carcinoma. It has been shown to be related to biphasic morphology.^[23,27]^ Comprehensive analysis provides a favorable basis for PD-L1 as an immunotherapy or targeted therapy for TFEB-associated renal cell carcinoma, but further studies are necessary. In terms of prognosis, some studies have showed that most TFEB-associated renal cell carcinoma exhibit inert behavior, and approximately 17% of the cases exhibit invasive behavior.^[13]^ Wyvekens et al^[10,28]^ also pointed out that TFEB-associated renal cell carcinoma seems to have a better prognosis. Seven patients with TFEB-associated renal cell carcinoma were followed-up by Rao et al During an average follow-up period of 31 months, no tumor recurrence, progression or metastasis occurred in any patient.^[29]^ To date, there are no factor to predict tumor invasiveness.^[30]^ Progression of TFEB-associated renal cell carcinoma is slow and inert. However, existing studies have limitations such as fewer cases and a short follow-up time. Additional cases and longer follow-up periods are required.

In summary, we reported a case of MIT family translocation renal cell carcinoma and review the relevant literature. TFEB-associated renal cell carcinoma translocation-fused alpha (MALAT1) and TFEB transcription factor genes. The morphology of these patients was related to the TFEB and showed a typical biphasic structure. TFEB-associated renal cell carcinoma was not marked as an epithelial immunohistochemical marker but was marked as melanocyte markers HMB45 and melan-A. IHC is an effective screening method for the TFEB protein, and FISH is the gold standard for diagnosis. Surgery is the primary treatment option. Targeted therapy and immunotherapy may be effective treatments for these conditions. Many studies have reported that TFEB-associated renal cell carcinoma has inert behavior.

## Author contributions

**Investigation:** Lin Li.

**Project administration:** Delin Yang.

**Resources:** Yitian Ou, Chao Zhang.

**Supervision:** Delin Yang.

**Validation:** Chengxing Xia, Delin Yang.

**Writing – original draft:** Yong Zhu.

## References

[1] Moch H Cubilla AL Humphrey PA. The 2016 WHO classification of tumours of the urinary system and male genital organs. Part A: renal, penile, and testicular tumours. Eur Urol. 2016;70:93–105. 26935559 10.1016/j.eururo.2016.02.029

[2] Inamura K. Translocation renal cell carcinoma: an update on clinicopathological and molecular features. Cancers (Basel). 2017;9:111. 28850056 10.3390/cancers9090111 PMC5615326

[3] Komai Y Fujiwara M Fujii Y. Adult Xp11 translocation renal cell carcinoma diagnosed by cytogenetics and immunohistochemistry. Clin Cancer Res. 2009;15:1170–6. 19228722 10.1158/1078-0432.CCR-08-1183

[4] Zhong M De Angelo P Osborne L. Dual-color, break-apart FISH assay on paraffin-embedded tissues as an adjunct to diagnosis of Xp11 translocation renal cell carcinoma and alveolar soft part sarcoma. Am J Surg Pathol. 2010;34:757–66. 20421778 10.1097/PAS.0b013e3181dd577e PMC7386799

[5] Caliò A Segala D Munari E. MiT family translocation renal cell carcinoma: from the early descriptions to the current knowledge. Cancers (Basel). 2019;11:1110. 31382581 10.3390/cancers11081110 PMC6721505

[6] Pei J Cooper H Flieder DB. NEAT1-TFE3 and KAT6A-TFE3 renal cell carcinomas, new members of MIT family translocation renal cell carcinoma. Mod Pathol. 2019;32:710–6. 30622287 10.1038/s41379-018-0191-7 PMC6486435

[7] Tsuda M Davis IJ Argani P. TFE3 fusions activate MET signaling by transcriptional up-regulation, defining another class of tumors as candidates for therapeutic MET inhibition. Cancer Res. 2007;67:919–29. 17283122 10.1158/0008-5472.CAN-06-2855

[8] Argani P Reuter VE Zhang L. TFEB-amplified renal cell carcinomas: an aggressive molecular subset demonstrating variable melanocytic marker expression and morphologic heterogeneity. Am J Surg Pathol. 2016;40:1484–95. 27565001 10.1097/PAS.0000000000000720 PMC5069163

[9] Zhan HQ Li ST Shu Y. Alpha gene upregulates TFEB expression in renal cell carcinoma with t (6;11) translocation, which promotes cell canceration. Int J Oncol. 2018;52:933–44. 29328409 10.3892/ijo.2018.4239

[10] Wyvekens N Rechsteiner M Fritz C. Histological and molecular characterization of TFEB-rearranged renal cell carcinomas. Virchows Arch. 2019;474:625–31. 30706129 10.1007/s00428-019-02526-8

[11] Inamura K Fujiwara M Togashi Y. Diverse fusion patterns and heterogeneous clinicopathologic features of renal cell carcinoma with t (6;11) translocation. Am J Surg Pathol. 2012;36:35–42. 22173116 10.1097/PAS.0b013e3182293ec3

[12] Zhan HQ Qin R Li YL. TFEB promotes BCL-2 expression by upregulating its promoter activity in the t (6;11) translocation renal cell carcinomas. Am J Transl Res. 2021;13:8804–18. 34539996 PMC8430107

[13] Caliò A Harada S Brunelli M. TFEB rearranged renal cell carcinoma. A clinicopathologic and molecular study of 13 cases. Tumors harboring MALAT1-TFEB, ACTB-TFEB, and the novel NEAT1-TFEB translocations constantly express PDL1. Mod Pathol. 2021;34:842–50. 33208882 10.1038/s41379-020-00713-6

[14] Saleeb RM Srigley JR Sweet J. Melanotic MIT family translocation neoplasms: expanding the clinical and molecular spectrum of this unique entity of tumors. Pathol Res Pract. 2017;213:1412–8. 28969862 10.1016/j.prp.2017.08.004

[15] Argani P Laé M Hutchinson B. Renal carcinomas with the t(6;11) (p21;q12): clinicopathologic features and demonstration of the specific alpha-TFEB gene fusion by immunohistochemistry, RT-PCR, and DNA PCR. Am J Surg Pathol. 2005;29:230–40. 15644781 10.1097/01.pas.0000146007.54092.37

[16] Sun G Chen J Liang J. Integrated exome and RNA sequencing of TFE3-translocation renal cell carcinoma. Nat Commun. 2021;12:5262. 34489456 10.1038/s41467-021-25618-z PMC8421377

[17] Martignoni G Pea M Gobbo S. Cathepsin-K immunoreactivity distinguishes MiTF/TFE family renal translocation carcinomas from other renal carcinomas. Mod Pathol. 2009;22:1016–22. 19396149 10.1038/modpathol.2009.58

[18] Williamson SR Grignon DJ Cheng L. Renal cell carcinoma with chromosome 6p amplification including the TFEB gene: a novel mechanism of tumor pathogenesis?. Am J Surg Pathol. 2017;41:287–98. 28009604 10.1097/PAS.0000000000000776

[19] Calió A Grignon DJ Stohr BA. Renal cell carcinoma with TFE3 translocation and succinate dehydrogenase B mutation. Mod Pathol. 2017;30:407–15. 27910947 10.1038/modpathol.2016.200

[20] Rao Q Williamson SR Zhang S. TFE3 break-apart FISH has a higher sensitivity for Xp11.2 translocation-associated renal cell carcinoma compared with TFE3 or cathepsin K immunohistochemical staining alone: expanding the morphologic spectrum. Am J Surg Pathol. 2013;37:804–15. 23598965 10.1097/PAS.0b013e31827e17cb

[21] Argani P Yonescu R Morsberger L. Molecular confirmation of t (6;11) (p21;q12) renal cell carcinoma in archival paraffin-embedded material using a break-apart TFEB FISH assay expands its clinicopathologic spectrum. Am J Surg Pathol. 2012;36:1516–26. 22892601 10.1097/PAS.0b013e3182613d8f PMC4441265

[22] Green WM Yonescu R Morsberger L. Utilization of a TFE3 break-apart FISH assay in a renal tumor consultation service. Am J Surg Pathol. 2013;37:1150–63. 23715164 10.1097/PAS.0b013e31828a69ae

[23] Smith NE Illei PB Allaf M. t (6;11) renal cell carcinoma (RCC): expanded immunohistochemical profile emphasizing novel RCC markers and report of 10 new genetically confirmed cases. Am J Surg Pathol. 2014;38:604–14. 24618616 10.1097/PAS.0000000000000203 PMC4370182

[24] Kuroda N Tanaka A Sasaki N. Review of renal carcinoma with t (6;11) (p21; q12) with focus on clinical and pathobiological aspects. Histol Histopathol. 2013;28:685–90. 23426439 10.14670/HH-28.685

[25] Napolitano G Di Malta C Esposito A. A substrate-specific mTORC1 pathway underlies Birt-Hogg-Dubé syndrome. Nature. 2020;585:597–602. 32612235 10.1038/s41586-020-2444-0 PMC7610377

[26] Zhang C Duan Y Xia M. TFEB mediates immune evasion and resistance to mTOR inhibition of renal cell carcinoma via induction of PD-L1. Clin Cancer Res. 2019;25:6827–38. 31383732 10.1158/1078-0432.CCR-19-0733

[27] Gupta S Argani P Jungbluth AA. TFEB expression profiling in renal cell carcinomas: clinicopathologic correlations. Am J Surg Pathol. 2019;43:1445–61. 31600176 10.1097/PAS.0000000000001307 PMC6788764

[28] Zhan HQ Wang CF Zhu XZ. Renal cell carcinoma with t (6;11) translocation: a patient case with a novel Alpha-TFEB fusion point. J Clin Oncol. 2010;28:e709–13. 20823414 10.1200/JCO.2010.30.3172

[29] Rao Q Liu B Cheng L. Renal cell carcinomas with t (6;11) (p21; q12): a clinicopathologic study emphasizing unusual morphology, novel alpha-TFEB gene fusion point, immunobiomarkers, and ultrastructural features, as well as detection of the gene fusion by fluorescence in situ hybridization. Am J Surg Pathol. 2012;36:1327–38. 22895266 10.1097/PAS.0b013e31825aafb5

[30] Caliò A Brunelli M Segala D. t (6;11) renal cell carcinoma: a study of seven cases including two with aggressive behavior, and utility of CD68 (PG-M1) in the differential diagnosis with pure epithelioid PEComa/epithelioid angiomyolipoma. Mod Pathol. 2018;31:474–87. 29052596 10.1038/modpathol.2017.144

